# Application of Matrix-Assisted Laser Desorption/Ionization Mass Spectrometry Imaging for Food Analysis

**DOI:** 10.3390/foods8120633

**Published:** 2019-12-02

**Authors:** Mizuki Morisasa, Tomohiko Sato, Keisuke Kimura, Tsukasa Mori, Naoko Goto-Inoue

**Affiliations:** Department of Marine Science and Resources, College of Bioresource Sciences, Nihon University, 1866 Kameino, Fujisawa, Kanagawa 252-0880, Japan; xx1mizu9xx@gmail.com (M.M.); sato310.volley@gmail.com (T.S.); mucunguiyou412@gmail.com (K.K.); mori.tsukasa@nihon-u.ac.jp (T.M.)

**Keywords:** MALDI-MS imaging, amino acids, lipids, neuropeptides, nutrition factor

## Abstract

Food contains various compounds, and there are many methods available to analyze each of these components. However, the large amounts of low-molecular-weight metabolites in food, such as amino acids, organic acids, vitamins, lipids, and toxins, make it difficult to analyze the spatial distribution of these molecules. Matrix-assisted laser desorption/ionization mass spectrometry (MALDI-MS) imaging is a two-dimensional ionization technology that allows the detection of small metabolites in tissue sections without requiring purification, extraction, separation, or labeling. The application of MALDI-MS imaging in food analysis improves the visualization of these compounds to identify not only the nutritional content but also the geographical origin of the food. In this review, we provide an overview of some recent applications of MALDI-MS imaging, demonstrating the advantages and prospects of this technology compared to conventional approaches. Further development and enhancement of MALDI-MS imaging is expected to offer great benefits to consumers, researchers, and food producers with respect to breeding improvement, traceability, the development of value-added foods, and improved safety assessments.

## 1. Introduction

Food ingredients contain a wide variety of nutritional components such as carbohydrates, proteins, peptides, lipids, minerals, vitamins, amino acids, and organic acids. In addition to the intentionally included ingredients, food can also contain contaminants such as pesticide residue, the residue of pharmaceuticals given to animals, mycotoxins, food additives, and carcinogenic substances introduced during food processing. Therefore, from the point of view of food safety, it is very important to verify all constituents in food. The diverse physical properties of these constituents require varied methods of analysis for detection, and these ingredients generally must first be purified for qualitative and quantitative analyses. Gas chromatography-mass spectrometry (GC-MS) and high-performance liquid chromatography-mass spectrometry (HPLC-MS) are commonly used to analyze food components. GC-MS is used for the detection and identification of volatile organic compounds such as amino acids, polyols, and vitamins, which are commonly derivatized [1]. HPLC-MS, however, is used to analyze higher molecular weight polar compounds [2,3]. Therefore, the application scope of these methods is wide and versatile. However, GC-MS and HPLC-MS are not well-suited for the analysis of the spatial distribution of compounds in food. In addition to the amounts of these compounds, determining their localization could provide useful information for food safety and for plant breeding and food processing applications. Conventional imaging techniques such as infrared spectrometry (IR) [4] and magnetic resonance imaging (MRI) [5] are also widely applied to food analysis. However, compounds from the spectrum are difficult to identify with IR, and MRI is not effective to identify substances with a low water content.

MS-based imaging utilizing the principle of matrix-assisted laser desorption/ionization (MALDI) is a relatively new imaging method for small metabolites [6,7,8], lipids [9,10,11,12], and proteins (peptides) [13,14,15]. MALDI-MS imaging is a two-dimensional analysis method that can detect intact molecules within tissue sections without requiring extraction, purification, separation, or labeling, and is the most applicable method owing to its ability to detect a wide range of molecules. The spatial resolution of MALDI-MS, depending on the laser radiation interval, can be set over a wide range of 5–200 μm, which is sufficient to obtain the molecular distribution from a single cell. Because MALDI-MS imaging can detect all molecules that undergo ionization, it has attracted significant attention as a non-target analysis method. Unlike conventional imaging methods such as immunohistochemistry, MALDI-MS imaging does not require the labeling of target molecules before analyses, making it one of the most powerful and convenient tools to screen molecules that show characteristic localization, especially in the medical field. The tandem mass spectrometry feature used directly on tissue sections allows for structure identification at a region of interest. In this review, we focus on recent applications of MALDI-MS imaging for food analyses, highlighting the great potential of this technique to improve quality control and food safety.

## 2. History of MALDI-MS Imaging Applications

The concept of MALDI-MS imaging was first introduced in the early 2000s. Caprioli et al. [16] performed direct ionization of proteins using rat pancreatic tissue sections, and Stoeckli et al. [17] demonstrated protein localization in the mouse cerebrum. At that time, the spatial resolution was still at a visual level (mm), but technological innovation improved this to 10 μm, and high-speed analysis with laser frequencies at 200–1000 Hz was realized in the 2010s [6]. These advances allowed for the capture of single cell-specific molecular distributions with MALDI-MS imaging.

MALDI-MS imaging was initially developed to analyze protein localization. At this time, there was a great demand for the detection of post-translational modifications according to a mass difference or the localization of hormone-like substances for which specific antibodies are difficult to obtain. However, this was not an easy task owing to quantitative limitations of these molecules. In contrast, lipids and very small molecules such as organic acids and nucleic acids were confirmed to be favorable molecules for detection with MALDI-MS imaging [18,19,20]. MALDI-MS imaging emerged in the medial field, especially in the field of cancer science to gain a better understanding of the metabolite dynamics during tumorigenesis [21], and has also been applied to the food field [22]. In food science, MALDI-MS imaging is now widely used to determine the localization of specific substances of interest such as sugars, amino acids, lipids, and polyphenols [23,24,25], or to screen non-target molecular dynamics in breeding or dosing applications. Thus, MALDI-MS imaging is used not only to clarify the localization of substances in food but also to ensure food safety.

MALDI-MS imaging has emerged as a valuable tool for the visualization of low-molecular-weight metabolites, but sometimes the matrix itself contributes to ionization interference. For this reason, other ionization methods such as desorption electrospray ionization mass spectrometry (DESI-MS) [26,27] and secondary ion mass spectrometry (SIMS) [28] are also used for MS imaging. DESI-MS [29] was introduced in 2004 as an ambient ionization method to directly ionize solid-phase samples at atmospheric pressure. One of the main advantages of DESI-MS is that since it does not require matrix spraying, it induces minimal damage to the sample without complicated matrix interference [30], thereby enabling the detection of minor components. Moreover, DESI-MS does not require the use of a special coating with indium thin oxide (ITO) to the slide glass, and the samples can be simultaneously applied to both imaging and histochemical analyses, allowing for accurate correlation analysis between molecular signatures and the histological state. In some cases, DESI-MS can ionize nonpolar compounds such as carotenes, which are abundantly present in plants and difficult to ionize with MALDI-MS. These properties have made DESI-MS a valuable tool for within-tissue detection of the spatial distribution of specialized metabolites [31], the visualization of plant metabolites, and investigations of their biological roles [32]. In contrast, the main advantage of SIMS is the ability to measure the spatial localization of molecules with high spatial resolution. MALDI imaging has a resolution of several tens of micrometers, whereas that of SIMS can be 200 nm or less. Although the samples used for SIMS also do not require any special surface treatment, the samples might be lost since SIMS can be a destructive analysis. Nevertheless, the improved resolution has now made it possible to image biomolecules at the sub-cellular level with this technique [28]. Those three ionization methods, MALDI, DESI, and SIMS, are the most used analytical imaging techniques in food sciences.

**Figure 1:**
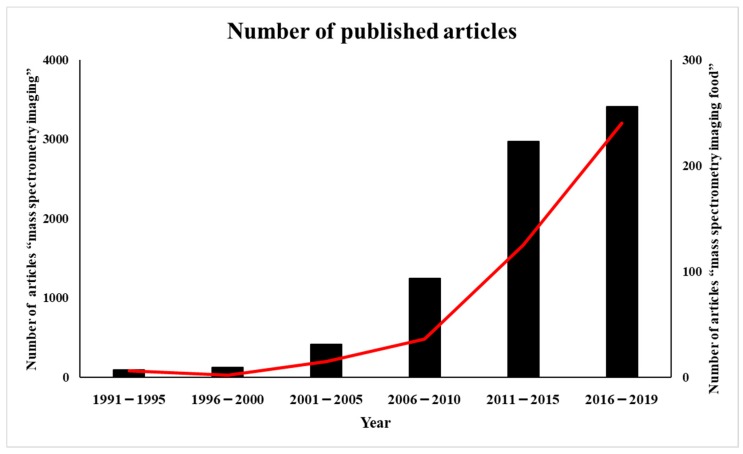
Publication trend (number of published articles) related to mass spectrometry imaging (black bars) and mass spectrometry imaging of food (red line).

The number of published articles with the key term “mass spectrometry imaging” in the PubMed database has gradually increased in the last 20 years. Only 90 papers on the topic were published up to 1995, whereas more than 3000 papers applying this technology have been published from 2016 to 2019. With respect to its application in food sciences, there were only 10 papers retrieved from a search with the key term “mass spectrometry imaging food” up to 2000. However, the number of papers doubled every 5 years thereafter, increasing to 15 in 2001–2005 and to 36 in 2006–2010; moreover, this number has been increasing rapidly in the last decade, with 240 papers reported since 2016 in this field. Many of these studies were aimed at using MS imaging to ensure the reliability of food and to increase the value of the food (Figure 1).

## 3. Sample Pretreatment for MALDI MS-Imaging

**Figure 2:**
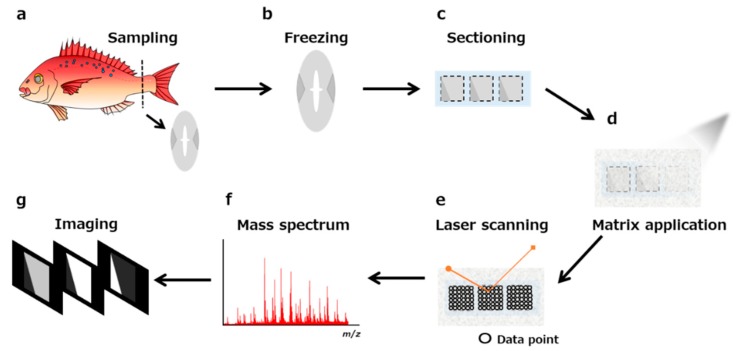
Scheme of matrix-assisted laser desorption/ionization mass spectrometry matrix-assisted laser desorption/ionization mass spectrometry (MALDI-MS) imaging. (**a**) Tissue sampling. (**b**) Preparation of a fresh-frozen sample. (**c**) Sectioning. (**d**) Application of the matrix. (**e**) Laser scanning. (**f**) Procurement of the mass spectrum. (**g**) Visualization of the ion distribution of molecules.

A schematic of the protocol for MALDI-MS imaging is shown in Figure 2. Here, we focus on the most important experimental steps such as sectioning, pretreatment of the section, and choice of matrix (e.g., 2,5-dihydroxybenzoic acid, α-cyano-4-hydroxycinnamic acid, 9-aminoacridine) and method for matrix application (e.g., spraying, deposition, and sublimation). To obtain useful results with MALDI-MS imaging, the sample pretreatment step is arguably the most important overall. In particular, the sample type, size, thickness, matrix, method of coating matrix, and other related factors need to be predetermined. Table 1 summarizes the recent applications of MALDI-MS imaging for food samples, demonstrating a wide variety of target molecules associated with equally diverse preparation methods. The optimal conditions for sample preparation need to be determined according to the sample. Here, we focus on the key aspects and related methods to prepare a sample to ensure reliable MALDI-MS imaging data.

### 3.1. Sample Storage

The biological tissue samples used for MALDI-MS imaging require storage at −80 °C to maintain the intact form and spatial organization of the biomolecules in the samples. The most favorable tissues for this purpose are fresh-frozen tissues, which can be prepared with various methods such as using powered dry ice, liquid nitrogen, or liquid nitrogen-chilled isopentane, among others [51]. In addition to frozen tissues, formalin-fixed, paraffin-embedded (FFPE) tissues sections could be applied to MALDI-MS imaging. However, FFPE tissues sections are completely stripped off lipophilic molecules after the deparaffinization step [52]; therefore, these sections have been adapted to detect proteins or peptides.

### 3.2. Embedding

Small-sized tissues and high-water content samples are hard to cut into appropriate sections without mounting. However, the use of typical embedding agents such as an optimal cutting temperature (OCT) compound must be avoided for samples destined for MALDI-MS imaging since the molecules derived from these agents can introduce ionization interference with respect to the biomolecules of interest [53]. Therefore, carboxymethyl cellulose (CMC) is recommended as the embedding material of tissue samples for MALDI-MS imaging, in addition to the use of 2% sodium CMC as an alternative embedding compound [54]. Khatib-Shahidi et al. [55] detected the drug and metabolite distributions in whole-body tissue sections at various time points following drug administration using CMC-embedded tissues. This was the first report indicating that CMC-embedded tissues can be used for MALDI-MS imaging. Especially, foods and/or plant with high water content sometimes need embedding to maintain their shape [24,36]. This approach is also applicable for the discovery of the localization of nutritional factors in plants.

### 3.3. Sectioning

The ionization efficiency is partly dependent on the thickness of the tissue section. In general, 5–20-μm-thick sections are prepared for the analysis of low-molecular-weight molecules. The use of thinner tissue sections (2–5 μm in thickness) are recommended for the analysis of high-molecular-weight molecules (3–21 kDa). We recommend the use of an ITO-coated glass slide for thaw-mounting of the sections because these transparent slides enable microscopic observation of the section after MALDI-MS imaging.

One of the major challenges of MALDI-MS imaging is maintaining the original shape of the tissue during the preparation of sections, which is particularly difficult when a section is created from fragile, and/or hard samples. High-quality sections can be made by creating a section using an adhesive film [56], which is suitable for attaching a tissue as a section, and is then attached to MALDI target plates or ITO-coated glass slides. However, this is associated with the severe drawback of reduced ionization efficiency compared to that obtained when using frozen sections on an ITO-coated glass slide [57]. There is also a method to transfer only the tissue from the film to the glass slide using an ultraviolet light source, but this requires a very advanced technique and specialized equipment [58]. Recently, Nakabayashi et al. [59] reported a technique to directly transfer a prepared section from a film to ITO-coated glass slides, which improves sample preparation for plant tissues with a high water content.

### 3.4. Sample Pretreatment (Washing, Digestion)

For protein imaging, it is essential to first wash out the lipophilic molecules on the tissue sections. The typical washing procedure is performed with a gradient of ethanol reagents. Finally, the sections are dried in a vacuum desiccator for 15 min [60]. For the detection of digested peptides, treatment with protease is also performed [61]. As mentioned, FFPE sections are used for protein and/or peptide detection [40]. In addition to FFPE sections, frozen sections could by applied for peptide imaging. Heijs et al. [62] conducted a comprehensive study of the mouse brain proteome using MALDI-MS imaging. With the treatment of complementary proteases, they were able to identify 5337 peptides from the MALDI-MS imaging results, corresponding to 1198 proteins. In addition to the common proteases such as trypsin, pepsin, and elastase [63], an enzyme for cutting *N*-linked glycans (peptide *N*-glycosidase F; PNGase F) has also been used to analyze post-translational modifications [64]. *N*-glycans play a fundamental role in many molecular and cellular processes and are established biomarkers of diseases [65,66]. MALDI-MS imaging with PNGase F—based tissue digestion has been successfully applied for the analysis of *N*-glycans in tissue microarrays [67]. For quantitative analysis, stable isotope-labeled internal standards are sprayed onto the sections [68].

### 3.5. Derivatization for Minor Targets

Ionized molecules are limited to abundant molecules and/or molecules with high ionization efficiency. Therefore, it is more challenging to detect minor components with conventional sample preparation. MALDI-MS imaging can be applied to overcome this problem through the development of a derivatization approach. Derivatization has long been used for quantitative analysis by GC-MS and HPLC-MS as it can enhance the ionization efficiency of molecules that are difficult to ionize. Due to the improved efficiency of derivatization, it has become a more common application in the field of MALDI. A representative example of such minor components is neurotransmitters. Derivatization converts low-molecular-weight endogenous primary amines into readily-ionized species of relatively high molecular weight, which enables the simultaneous imaging of tyrosine, tryptamine, tyramine, phenethylamine, dopamine, 3-methoxytryramine, serotonin, gamma-aminobutyric acid (GABA), and glutamate, along with other endogenous primary amines, in a single MALDI-MS image. These technologies have now made it possible to measure the distributions of a wide range of primary amines with high resolution [69]. We must also take into account the possibility of the lateral drift of molecules due to the reagents. To prevent the dispersion of molecules with these solvent, a previous study used an automatic sprayer to fix spray conditions.

### 3.6. Matrix Selection

The selection of an appropriate matrix is undoubtedly the most important step in optimizing the MALDI-MS conditions for a given application. In particular, the purity, concentration, and solubility are important considerations to adapt to the chemical properties of the target compounds. In addition to the type of matrix, there are various methods to apply a matrix to the sections. The commonly used matrices for MALDI-MS imaging are listed in Table 2. The matrix plays a central role in MALDI-MS soft ionization [70,71], in which the biomolecules are softly ionized in the co-crystal with the matrix, which absorbs the laser beam energy to protect the biomolecules from the disruptive energy. Protonated ions ([M + H]^+^) or deprotonated ions ([M − H]^−^) are generally detected, and sodium adduct ions ([M + Na]^+^) and potassium adduct ions ([M + K]^+^) are often observed in the analyses of biological samples. Thus, the choice of an appropriate matrix is essential to obtain meaningful biomolecule images [72,73]. The choice of a matrix used for MALDI-MS imaging depends on the chemical properties and mass range of the analytes of interest. In general, 2,5-dihydroxybenzoic acid and 9-aminoacridine are considered suitable materials for lipids and small metabolites.

### 3.7. Matrix Coating

In MALDI-MS imaging, the analytes must be co-crystallized with the matrices to be appropriately ionized. Therefore, we must minimize the crystal size of matrices that influence the spatial resolution of molecules. There are various methods available to coat the matrix onto the section, including deposition, spraying, and sublimation. The method of matrix application also influences the analyte extraction efficiency. Spraying is the most frequently used method in MALDI-MS imaging [74], and can coat the entire tissue section with relatively small crystals that are homogeneously dispersed within a short time without any special equipment. However, this method requires skillful operation to manipulate the numerous parameters with a manually operated airbrush.

To overcome the problem of reproducibility associated with spraying conditions, an automatic matrix sprayer system can be used, which was previously reported to result in approximately double the number of metabolites detected compared to that detected when using the manual airbrush method [75]. Thus, the use of an automated system could enhance the sensitivity of molecule detection. An explanation for this effect is that the TM-Sprayer, an automatic matrix sprayer system, sprays the solution at a high temperature, thereby forming more homogeneous matrix crystals on the tissue than possible with an airbrush [75,76].

## 4. Localization of Small Metabolites in Foods

MALDI-MS imaging is now becoming widely applied for localization analysis of molecules in food, which is one of the simplest methods of MALDI-MS imaging, revealing characteristic substances with various approaches. Some of these studies have focused on the localization of nutritional factors. GABA plays various beneficial roles such as controlling plant growth and resistance to oxidative stress, and is one of the most widely recognized ingredients of functional foods. Although the precise localization of GABA had not been clarified because of the lack of a specific probe, MALDI-MS imaging, for the first time, enabled visualization of its characteristic localization in the seeds of eggplants. The determination of the fine localization of GABA in crops would be useful for plant breeding and food processing [23]. In the same manner, small molecules in plant tissues were previously analyzed to identify these localizations [34,41]

Fruit taste is largely dependent on the balance and content of sweetness and sourness. Sweetness and acidity are determined by sugar (especially glucose, fructose, and sucrose) and organic acid (citric acid and malic acid), respectively; however, their spatial localization remains undetermined. The application of MALDI-MS to strawberries revealed different substances in each part of the fruit. For example, glucose and fructose were distributed throughout the strawberry section, whereas sucrose was predominantly distributed on the top side of the cortical tissue and in the vascular bundles. This study further showed that the sweetness of each part of the strawberry differs according to the constituent substance of that part. Thus, MALDI-MS imaging not only clarified the characteristic patterns of metabolism in strawberries but also provided reference information to guide future strawberry breeding improvements [24]. 

Solanine and chaconine, contained in potatoes, are steroidal alkaloid glycosides that are known to be toxic to the human body; consuming large amounts of these toxins results in typical symptoms of nausea and abdominal pain. The total amount of toxins contained in potato buds has been determined by separation analysis methods such as liquid chromatography, but there are few methods available to visualize their distribution in foodstuffs. Tissue distribution determined by MALDI-MS imaging revealed the presence of solanine and chaconine on the bud and perimeter of persimmons. This approach therefore provides visually accessible data for experts and for consumers, who can intuitively understand the location of targeted components [39].

Capsaicin is a pungent ingredient of capsicum fruits, which can be roughly divided into the pericarp, placenta, and seed regions. MALDI-MS imaging demonstrated that capsaicin was localized to the surface of the placenta and pericarp but was scarcely present in the seed. From these results, it was considered that capsaicin is likely present in the pericarp to protect the fruit from external enemies, and that a large amount of capsaicin is produced on the surface of the placenta to protect the seed [25].

## 5. Breed Improvement with MALDI-MS Imaging-Based Localization Analysis

In addition to issues of food safety and quality control, identifying the distribution and contents of various food components can also provide valuable information for plant breeding and aquaculture. Rice is a crop that has undergone many breed improvements to increase its nutritional quality. Zaima et al. [36] used MALDI-MS imaging to demonstrate how the localization of substances (metabolites and minerals) in rice can be used to investigate changes occurring with breed improvement. In this study, the researchers detected lysophosphatidylcholine within the endosperm, α-tocopherol within the scutellum, and phosphatidycholine, ɤ-oryzanol, and phytic acid in the bran. In particular, this was the first study to analyze the characteristic distributions of α-tocopherol, ɤ-oryzanol, and phytic acid, as three important nutritional components. In addition, the localization of arginine was different as a result of breeding, with various distribution patterns among rice types and growth stages.

Anthocyanin is present in various forms with respect to the glycosides. The localization of seven types of anthocyanin monoglycosides and two types of anthocyanin diglycosides was determined in black rice and blueberries, demonstrating that the distribution varied among the different structures of the foods [38]. Furthermore, using MALDI-MS imaging, which does not require extraction and concentration, the researchers could detect fragile molecules including two types of anthocyanin pentosides, which are difficult to detect by conventional HPLC owing to their unstable structure.

**Figure 3:**
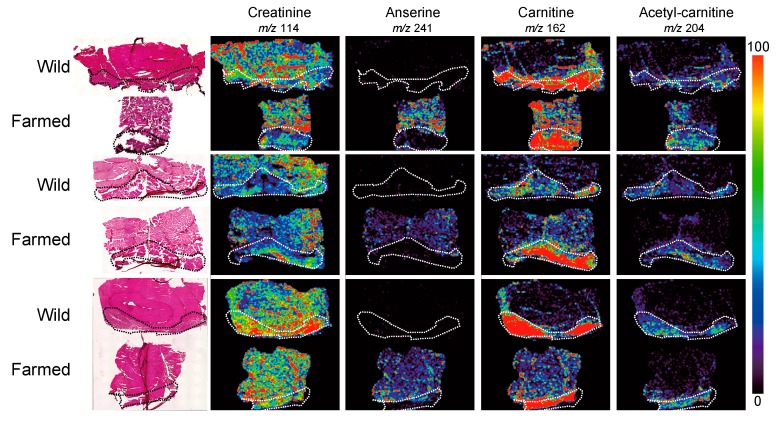
Matrix-assisted laser desorption/ionization mass spectrometry (MALDI-MS) imaging results of creatinine, anserine, carnitine, and acetyl carnitine distributions in wild and farmed red sea bream. Dotted outlines show the red muscle regions. Modified and reprinted with permission from [46].

MALDI-MS imaging has also been applied in the field of aquaculture. Sroyraya et al. [35] visualized the metabolites of the eyestalk of the blue swimming club, and Piyachat et al. [13] showed the distribution of neuropeptides in shrimp. We also analyzed the metabolic molecules of wild and farmed red sea bream to identify species-specific metabolic differences. Because the complete genome sequence of red sea bream is not yet complete, it has been very difficult to find molecular markers of wild and farmed fish for their respective identification. However, using MALDI-MS imaging, we were able to discover some lipid molecules that differed between wild and farmed fish. Furthermore, the specific distribution of anserine, a well-known nutrition factor, in farmed red sea bream could be detected (Figure 3) [46].

Since the discovery of bovine spongiform encephalopathy, consumers have been faced with doubts about the safety of beef; thus, the geographical origin of meat is very important information that will influence consumer choice. Gene analysis and stable isotope analysis are applied to trace a beef sample to its geographic origin, but time-consuming procedures have made it difficult to distinguish individuals of the same breed according to location. Therefore, we applied MALDI-MS imaging to identify molecular markers of geographical origin based on principal component analysis in three independent beef tissue samples, which exhibited different geographical origins, and some key molecules were identified that could be used to classify their origin [37].

## 6. Recent Developments and Future Perspectives of Mass Spectrometry Imaging

**Figure 4:**
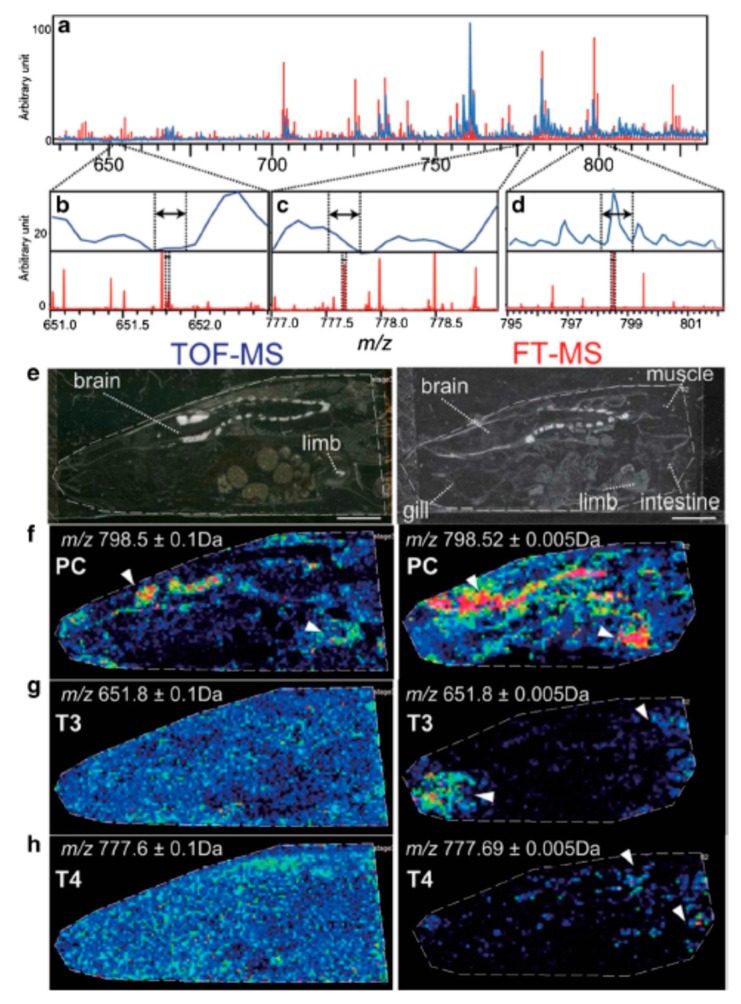
Time-of-flight mass spectrometry (TOF-MS) and Fourier-transform ion cyclotron resonance (FT-ICR) MS imaging results. Mass resolution was substantially higher in the FT-ICR mass spectrum, allowing for the visualizing of the specific localization of T3 and T4 in amphibian larvae. Modified and reprinted with permission from [77]. Mass spectra of tissue sections (**a**–**d**). Optical images of the tissue sections (**e**) and ion images of phosphatidylcholine, T3, and T4, respectively (**f**–**h**).

Time-of-flight (TOF) technology is widely used for MS imaging, as this can effectively separate ionized accelerated molecules according to their mass-to-charge ratio (*m/z*). However, the mass resolution of TOF-MS is still not sufficient for a wide detection range since the single image is reconstructed at a resolution of ±0.1–0.2 Da, which might result in the overlap of multiple molecules within one constructed image. However, recent technological developments succeeded in achieving a higher mass resolution of approximately ±0.01 Da using Fourier-transform ion cyclotron resonance (FT-ICR) MS. For example, TOF-MS could not reveal the characteristic localization of thyroxine hormone (T3) in amphibian larvae owing to multiple mixed molecules including phospholipids in the vicinity of the molecular weight of the target. However, when the same sample was analyzed with FT-ICR MS, T3 and T4-specific peaks were observed (Figure 4) [77].

To obtain high spatial resolution, new matrix coating methods have also been established. Shuai et al. developed an electric field-assisted scanning-spraying matrix coating system to deposit the matrix on tissue with crystal sizes of <10 μm [78]. This system can easily generate homogenous and small matrix crystals on the tissue. It also enhanced the detection and imaging quality of tissue small molecule metabolites (<500 Da).

Recently, new ionization technology for higher ionization efficiency has been reported. Laser ablation DART imaging mass spectrometry was performed to detect the spatial distributions of highly volatile compounds in cross sections of *Coffea arabica* beans [79]. Moreover, a novel MS-based imaging platform was developed by integrating a new subatmospheric pressure MALDI source. This apparatus could perform in situ *N*-glycan imaging analysis with high resolution [80]. In 2019, Niehaus et al. developed a new ion source for transmission-mode geometry MALDI-MS imaging [81]. It can provide molecular information with a pixel size of 1 μm and smaller. This method could thus be a valuable new tool for research in cell biology.

MALDI-MS imaging generates an enormous amount of data. Therefore, diversification of analysis methods such as cloud-based analysis (e.g., SCiLS, https://scils.de/) will be needed in the near future to handle such big data for convenient interpretation. These data platforms could also perform multivariate statistical analyses, which could lead to meaningful data interpretation for researchers. In fact, datasets of unlimited size can be visualized and multivariate statistical analyses can be performed for detailed interpretation [82,83]. The data format imzML allows for the flexible and efficient exchange of MS imaging data between different instruments and data analysis software [84]. A number of software tools are available and many more are being adapted to imzML. (https://ms-imaging.org/wp/imzml/)

## 7. Conclusions

MALDI-MS imaging is a valuable tool to visualize food compounds and identify not only the nutritional content but also the geographical origin of the food for improved traceability, food safety, and breed enhancement, among other applications. We anticipate that MALDI-MS imaging will be used extensively in the food industry in the near future. However, certain challenges of this technology will need to be overcome, including the limited detection of molecules present at low concentrations or ionization efficiency. Therefore, further improvements to the method and/or new developments in the equipment should be a research focus to enable the sensitive detection of these molecules.

## Figures and Tables

**Table 1:** Overview of the application of matrix-assisted laser desorption/ionization mass spectrometry (MALDI-MS) imaging for food science and related fields.

Sample	Target Molecules	Sample Preparation Sample Type Thickness Embedding	Matrices	Reference
Soya leaf, stem	Mesotorione, azoxystrobin	Freeze-drying - -	CHCA	[8]
Strawberry fruit skin	Sucrose, fructose, glucose, citric acid	Fresh 0.2–0.5 mm with a sharp utility knife -	DHB	[33]
Wheat grain	Glucose-6-phosphate, sucrose	Frozen - Ice	CHCA	[7]
Wheat stem	Oligosaccharides	Freeze-drying 50 μm -	CHCA	[34]
Ginger rhizome (*Zingiber officinale*)	6-gingerol, monoterpene	Fresh 0.2 mm -	-	[6]
Eggplant	GABA, nicotinic acid, arginine, 2-aminobenzoic acid, citric acid, saccharides	Frozen 14 μm -	DHB	[23]
Blue swimming crab (*Portunus pelagicus*)	Phospholipids, triacylglycerols	Frozen 14 μm 2% CMC	DHB	[35]
Rice seed	Phospholipids, α-tocopherol, arginine, ɤ-oryzanol, phytic acid	Frozen 8 μm with adhesive film (Kawamoto method) 2% CMC	DHB	[36]
Beef meat	Lipids	Frozen 8 μm -	DHB	[37]
*Penaeus monodon*	Neuropeptides	Frozen 5 μm Paraffin	CHCA	[13]
*Capsicum annuum*	Capsaicin	Frozen 70 μm -	CHCA	[25]
Black rice seed	Lysophosphatidylcholine, phosphatidylcholine, anthocyanins	Frozen 10 μm with adhesive film (Kawamoto method) 2% CMC	DHB	[38]
*Camelina sativa* seed transgenic	Lipids	Frozen 30–50 μm 10% gelatin	DHB	[10]
Potato (*Solanum tuberosum* L.)	α-solanine, α-chaconine	Frozen - -	CHCA	[39]
Wheat (*Triticum aestivum* L.)	Polysaccharides	Frozen 60 μm -	DHB	[40]
Tomato fruit (*S. lycopersicum* L.)	Organic acid, amino acid nucleotides, caffeic acid	Frozen 10 μm OCT compound	DHB, 9-AA	[41]
Rice (*Oryza sativa* L.)	Cytokinin, abscisic acid	Frozen 50 μm Ice	CHCA	[42]
Cucumber	Triterpenes	Frozen 50 μm -	-	[43]
Maize seed (*Zea mays*)	Triacylglycerols, amino acids	Frozen 10 μm -	DAN, DHB, 9-AA	[44]
Oilseed rape (*Brassica napus*)	Lipids	Frozen 30 μm -	DHB	[45]
Strawberry	Anthocyanins, sugars, organic acids	Frozen 80 μm 2% CMC	DHB	[24]
Red sea bream (*Pagrus major*)	Lipids	Frozen 15 μm -	DHB	[46]
Grain (*Triticum aestivum* L.)	Hemicelluloses	Frozen 80 μm -	DMA, DHB	[47]
Ham	Peptide	Frozen 12 μm -	CHCA	[48]
Apple	Soluble carbohydrate	Fresh 20 μm -	CHCA, DHB	[49]
Nightshades	Alkaloids	Frozen 40 μm Ice	DHB	[50]
Pork chop	Lipids	Frozen 10 μm -	CHCA, DHB	[9]

GABA, gamma-aminobutyric acid; CMC, carboxymethyl cellulose; OCT, optimal cutting temperature; CHCA, α-cyano-4-hydroxycinnamic acid; DAN, 1,5-diaminonaphthalene; DHB, 2,5-dihydroxybenzonic acid; DMA, *N,N*-dimethylaniline; 9-AA, 9-aminoacridine.

**Table 2:** Common matrix types used in matrix-assisted laser desorption/ionization mass spectrometry (MALDI-MS) imaging according to the sample type.

Matrices	Sample
9-Aminoacridine (9-AA)	lipids, metabolites
Sinapinic acid (SA)	peptides, proteins
Nicotinic acid (NA)	nucleotide
2,5-Dihydroxybenzoin acid (DHB)	lipids, glycopeptide, polymer
3-Amino-4-hydroxybezoic acid (AHBA)	glycan
α-Cyano-4-hydroxycinnamic acid (CHCA)	peptides, proteins
1,5-Diaminonapthalene (DAN)	lipids
t3-Indolacrylic acid (IAA)	polymer, aromatic
2-(4-Hydroxyphenylazo)-benzoic acid (HABA)	polymer
3-Aminoquinoline (3AQ)	glycan
Picolinic acid (PA)	nucleotide
Anthranilic acid (ANA)	nucleotide
3-Hydroxypicolinic acid (3HPA)	nucleotide
5-Chlorosalycilic acid (5CSA)	polymer
Dihydroxyacetone phosphate (DHAP)	lipids, glycan
